# Genome-wide identification and expression analysis of sucrose nonfermenting-1-related protein kinase (*SnRK*) genes in *Triticum aestivum* in response to abiotic stress

**DOI:** 10.1038/s41598-021-99639-5

**Published:** 2021-11-18

**Authors:** Shefali Mishra, Pradeep Sharma, Rajender Singh, Ratan Tiwari, Gyanendra Pratap Singh

**Affiliations:** ICAR-Indian Institute of Wheat and Barley Research, Karnal, India

**Keywords:** Computational biology and bioinformatics, Molecular biology

## Abstract

The SnRK gene family is a key regulator that plays an important role in plant stress response by phosphorylating the target protein to regulate subsequent signaling pathways. This study was aimed to perform a genome-wide analysis of the *SnRK* gene family in wheat and the expression profiling of SnRKs in response to abiotic stresses. An in silico analysis identified 174 *SnRK* genes, which were then categorized into three subgroups (SnRK1/2/3) on the basis of phylogenetic analyses and domain types. The gene intron–exon structure and protein-motif composition of *SnRKs* were similar within each subgroup but different amongst the groups. Gene duplication and synteny between the wheat and *Arabidopsis* genomes was also investigated in order to get insight into the evolutionary aspects of the TaSnRK family genes. The result of *cis*-acting element analysis showed that there were abundant stress- and hormone-related *cis*-elements in the promoter regions of 129 *SnRK* genes. Furthermore, quantitative real-time PCR data revealed that heat, salt and drought treatments enhanced TaSnRK2.11 expression, suggesting that it might be a candidate gene for abiotic stress tolerance. We also identified eight microRNAs targeting 16 *TaSnRK* genes which are playing important role across abiotic stresses and regulation in different pathways. These findings will aid in the functional characterization of TaSnRK genes for further research.

## Introduction

Bread wheat (*T. aestivum* L.) is a major cereal crop and an important source of carbohydrates and protein in the human diet, accounting for 20% of daily calorie consumption. The most significant economic feature is grain yield, which is influenced by a variety of biotic and abiotic stressors. By 2050, it is expected that the demand for wheat will increase by 60%^1^. Plants use a variety of molecular defence mechanisms to deal with abiotic stresses such as salt, drought, and heat. Plants respond to environmental stresses in two ways: gene expression regulation and protein modification^2^. Protein kinase-mediated phosphorylation and dephosphorylation are important in protein modification^3^. SnRKs (Sucrose nonfermenting 1 (SNF1)-related protein kinases) are a group of protein kinase genes that have a role in a variety of physiological activities^4^. Based on their sequence similarities and gene architectures, plant SnRKs may be split into three subfamilies: SnRK1, SnRK2, and SnRK3^4,5^. The SnRK1 subfamily has a highly conserved N-terminal protein kinase (Pkinase) domain, which is similar to SNF1 in yeast and AMPKs in mammals^6^. The SnRK2 and SnRK3 subfamilies are unique to plants, and both show more variability than the SnRK1 subfamily members in terms of plant diversity. A conserved P kinase domain and a C-terminal variable adjusting domain are found in members of the SnRK2 family^7^. SnRK3, known as CIPK (CBL-interacting protein kinases), also has conserved N-terminal protein kinase domains and NAF domains, as well as PPI domains at the C-terminus^8,9^.

Plant cells respond to starvation and metabolic stress through the SnRK1 family of genes. Catalytic components of heterotrimeric complexes, SnRK1 kinases interact with two additional subunits^10^. SnRK1 was shown to be involved in the stimulation of sucrose synthase expression and had a key function in carbohydrate metabolism control in *S. tuberosum*^11^. Low energy stress (e.g., darkness and hypoxia) may also cause SnRK1 nuclear translocation and subsequent induction of SnRK1 target genes, allowing for the replenishment of cellular energy for plant development^12^. SnRK1 genes were also shown to be hubs in a network of signalling pathways that included cell cycle control, pathogen responses, and meristem formation, as reported earlier^13^.

The SnRK2 genes, on the other hand, play a significant role in plants' responses to abiotic stressors, particularly osmotic and salt stress. In *A. thaliana*, for example, SnRK2.10 phosphorylates multiple target genes, including the dehydrins ERD10 and ERD14, to deal with osmotic stress^14^. In *N. tabacum*, SnRK2.1 regulates salt tolerance positively^15,16^. SnRK2 subfamily genes in *A. thaliana* may be divided into three groups: ABA-independent kinases, genes responsive to drought stress, and kinases that are substantially activated by ABA^7,17^. The most comprehensive study on ABA-dependent group 3 kinases is now underway. For example, one of the SnRK2 family genes, *AtSnRK2.6* (OST1), is involved in the ABA signalling pathway in stomatal guard cells, and OST1 protein stability may be regulated by the E3-ubiquitin ligase HOS15 in Arabidopsis to reduce ABA signal sensitivity^18,19^.

CBL (calcium sensor calcineurin B-like proteins)-interacting protein kinases (CIPKs), also known as SnRK3 kinases, play an important role in plant stress tolerance^4,20^. The SOS (salt excessively sensitive) system, for example, was the first CBL-CIPK route found in *A. thaliana*. SOS3 (AtCBL4) on the cell membrane recognised the calcium signal created by salt stress, and then SOS3 joined with SOS2 (AtCIPK24) to phosphorylate SOS1 (Na^+^/H^+^ antiporter) to remove excess Na^+^ from root cells^21,22^. MdCIPK13 and MdCIPK22 also improved salt and drought tolerance in apple by phosphorylating the sucrose transporter MdSUT2.2^23,24^. In *B. napus*, overexpression of BnCBL1-BnCIPK6 may improve high salinity tolerance and low potassium tolerance^25^. Finally, growing data has highlighted the relevance of SnRKs function in nutrient consumption and stress response, and researchers may be able to increase plant stress resistance by genetic modification of these genes.

The importance of *SnRK* genes function in nutrient consumption and stress response is becoming clearer, and researchers may be able to improve plant stress resistance by genetically modifying these genes. However, there is no information available on *SnRK* genes in wheat, one of the most important cereals. In the current study, we identified 174 *SnRK* gene family members in the genome of *Triticum aestivum*. We also evaluated their evolutionary relationships, gene architecture, protein motifs, chromosomal position, and *cis*-elements in promoter regions in a systematic way. The differential expression of the *SnRK* genes under abiotic stress was also investigated using qRT-PCR. The results obtained in this study provide important insights into the molecular pathways that underpin stress tolerance and molecular breeding.

## Results

### Identification of SnRK genes of wheat

A total of 174 *SnRK* genes were identified and further subdivided into three categories: SnRK 1, SnRK 2 and SnRK3 which have 14, 65 and 95 genes, respectively (Supplementary Table S1). The number of SnRK gene in wheat is higher than other plant species e.g. *A. thaliana* and rice. It may be due to hexaploid nature of wheat genome. Each protein's longest amino acid sequence was chosen for further investigation. The parameters of the gene characteristics including Gene IDs, amino acid length, molecular weights (MW), isoelectric points (pI), grand average of hydropathicity (GRAVY) and sub-cellular localization were analysed (Table 1). The molecular weight of all these TaSnRK proteins spans from 26.8 to 146.1 kDa, while the amino acid length spans from 239 to 1335. The isoelectric point of SnRKs family ranged from 4.3 to 9.4 indicating basic nature of proteins. The hydrophobicity and hydrophilicity are revealed by the GRAVY scores. SnRK proteins from *T. aestivum*, on the other hand, show a negative GRAVY score, implying that they are hydrophilic. The degree of hydrophilicity, on the other hand, is proportionate to the increased variability. According to the expected subcellular localization data, most of the *TaSnRK* genes are expressed in the nucleus and cytoplasm, followed by chloroplast and mitochondria (Table 1).

**Table 1:** List of the identified TaSnRK family members in *T. aestivum.*

Gene id	No. of amino acids	Mol. weight	Theoretical pI	Aliphatic index	(GRAVY)	localization	Domains
TaSnRK1.1	503	57,281.36	8.77	93.62	− 0.266	cyto	PKinase, UBA, KA1
TaSnRK1.2	500	56,961.98	8.77	94.18	− 0.268	cyto	PKinase, UBA, KA1
TaSnRK1.3	500	56,961.98	8.77	94.18	− 0.268	cyto	PKinase, UBA, KA1
TaSnRK1.4	503	57,492.46	8.58	90.89	− 0.362	cyto	PKinase, UBA, KA1
TaSnRK1.5	503	57,492.46	8.58	90.89	− 0.362	cyto	PKinase, UBA, KA1
TaSnRK1.6	503	57,476.47	8.58	91.29	− 0.352	cyto	PKinase, UBA, KA1
TaSnRK1.7	438	50,268.93	8.7	89.5	− 0.362	chlo	PKinase, UBA, KA1
TaSnRK1.8	509	58,194.2	8.65	89.27	− 0.316	cyto	PKinase, UBA, KA1
TaSnRK1.9	513	58,709.82	8.55	89.71	− 0.312	cyto	PKinase, UBA, KA1
TaSnRK1.10	509	58,250.26	8.55	89.84	− 0.318	cyto	PKinase, UBA, KA1
TaSnRK1.11	441	50,784.85	6.33	97.01	− 0.207	cyto	PKinase, UBA, KA1
TaSnRK1.12	466	53,389.7	6.86	97.04	− 0.222	vacu	PKinase, UBA, KA1
TaSnRK1.13	512	58,436.59	8.65	95.18	− 0.282	cyto	PKinase, UBA, KA1
TaSnRK1.14	516	58,901.13	8.53	95.58	− 0.292	cyto	PKinase, UBA, KA1
TaSnRK2.1	357	40,939.6	5.55	77.84	− 0.522	cysk	PKinase
TaSnRK2.2	357	40,911.55	5.55	77.31	− 0.529	cyto	PKinase
TaSnRK2.3	357	40,911.55	5.55	77.31	− 0.529	cyto	PKinase
TaSnRK2.4	394	44,210.97	6.15	73.3	− 0.481	cyto	PKinase
TaSnRK2.5	391	43,969.68	6.15	72.84	− 0.499	cyto	PKinase
TaSnRK2.6	391	43,927.6	6.15	72.33	− 0.511	cyto	PKinase
TaSnRK2.7	363	42,176.97	5.86	76.5	− 0.588	cysk	PKinase
TaSnRK2.8	363	42,096.89	5.77	76.5	− 0.562	cysk	PKinase
TaSnRK2.9	363	42,177.99	5.86	77.02	− 0.574	cysk	PKinase
TaSnRK2.10	342	38,609.93	5.43	83.6	− 0.431	cyto	PKinase
TaSnRK2.11	342	38,548.93	5.73	83.6	− 0.424	cyto	PKinase
TaSnRK2.12	342	38,663.04	5.52	83.89	− 0.435	cyto	PKinase
TaSnRK2.13	342	38,793.3	5.59	87.13	− 0.246	cyto	PKinase
TaSnRK2.14	342	38,847.35	5.66	87.43	− 0.249	cyto	PKinase
TaSnRK2.15	343	38,998.52	5.47	84.34	− 0.278	cyto	PKinase
TaSnRK2.16	341	38,664.29	5.45	89.97	− 0.208	cyto	PKinase
TaSnRK2.17	353	40,000.81	5.53	90.79	− 0.201	cyto	PKinase
TaSnRK2.18	353	40,014.88	5.62	90.79	− 0.197	cyto	PKinase
TaSnRK2.19	364	40,585.43	4.89	89.73	− 0.185	cysk	PKinase
TaSnRK2.20	363	40,659.53	4.89	88.65	− 0.217	cyto	PKinase
TaSnRK2.21	360	40,327.2	4.94	90.44	− 0.191	cyto	PKinase
TaSnRK2.22	330	37,143.13	4.68	88.64	− 0.261	cysk	PKinase
TaSnRK2.23	239	26,754.3	4.38	88.54	− 0.281	cyto	PKinase
TaSnRK2.24	361	40,631.17	4.8	88.86	− 0.271	cysk	PKinase
TaSnRK2.25	366	41,590.39	4.87	86.83	− 0.315	cysk	PKinase
TaSnRK2.26	366	41,590.39	4.87	86.83	− 0.315	cysk	PKinase
TaSnRK2.27	366	41,590.39	4.87	86.83	− 0.315	cysk	PKinase
TaSnRK2.28	388	42,192.41	6.76	87.96	− 0.164	chlo	PKinase
TaSnRK2.29	445	48,359.5	8.93	81.98	− 0.326	chlo	PKinase
TaSnRK2.30	448	48,647.72	8.89	80.78	− 0.343	nucl	PKinase
TaSnRK2.31	425	47,200.17	6.43	81.22	− 0.185	cyto	PKinase
TaSnRK2.32	625	70,055.65	5.36	88.96	− 0.26	cyto	PKinase
TaSnRK2.33	626	70,584.46	5.77	92.41	− 0.267	cyto	PKinase
TaSnRK2.34	623	70,148.93	5.73	92.23	− 0.261	E.R., golg	PKinase
TaSnRK2.35	341	38,664.29	5.45	89.97	− 0.208	cyto	PKinase
TaSnRK2.36	481	53,582.27	6.13	84.68	− 0.299	cyto	PKinase
TaSnRK2.37	481	53,597.33	6.13	84.89	− 0.289	cyto	PKinase
TaSnRK2.38	486	53,709.27	5.75	83.85	− 0.264	cyto	PKinase
TaSnRK2.39	486	53,751.31	5.75	84.05	− 0.265	cyto	PKinase
TaSnRK2.40	1332	145,867.36	8.09	99.62	− 0.042	plas	PKinase
TaSnRK2.41	1335	146,114.44	8.01	99.03	− 0.049	plas	PKinase
TaSnRK2.42	1335	146,061.45	8.01	99.03	− 0.051	cyto	PKinase
TaSnRK2.43	1027	114,149.62	5.4	82.56	− 0.418	cyto	PKinase
TaSnRK2.44	1027	114,193.62	5.44	82.94	− 0.424	cyto	PKinase
TaSnRK2.45	1027	114,053.56	5.42	83.6	− 0.409	cyto	PKinase
TaSnRK2.46	525	60,273.36	6.21	73.94	− 0.65	nucl	PKinase
TaSnRK2.47	527	60,480.56	6.21	73.11	− 0.654	nucl	PKinase
TaSnRK2.48	525	60,258.4	6.12	75.05	− 0.633	nucl	PKinase
TaSnRK2.49	539	61,557.02	6.1	72.6	− 0.588	nucl	PKinase
TaSnRK2.50	542	61,867.29	6.03	71.13	− 0.62	nucl	PKinase
TaSnRK2.51	538	61,455.88	6.1	71.65	− 0.608	nucl	PKinase
TaSnRK2.52	564	65,620.17	5.75	67.13	− 0.885	cyto	PKinase
TaSnRK2.53	564	65,294.61	5.75	67.11	− 0.878	cyto	PKinase
TaSnRK2.54	562	65,136.4	5.78	67.54	− 0.867	cyto	PKinase
TaSnRK2.55	462	53,394.1	8.27	72.23	− 0.704	chlo	PKinase
TaSnRK2.56	462	53,394.1	8.27	72.23	− 0.704	chlo	PKinase
TaSnRK2.57	462	53,424.13	8.27	72.23	− 0.705	nucl	PKinase
TaSnRK2.58	558	63,782.63	5.7	75.04	− 0.659	cyto	PKinase
TaSnRK2.59	558	63,919.73	5.71	74.16	− **0.67**	cyto	PKinase
TaSnRK2.60	558	63,901.75	5.71	75.56	− 0.658	cyto	PKinase
TaSnRK2.61	606	68,445.68	7.71	78.27	− 0.581	chlo	PKinase
TaSnRK2.62	948	104,031.17	6.71	71.4	− 0.579	nucl	PKinase
TaSnRK2.63	946	103,753.05	6.85	72.38	− 0.553	nucl	PKinase
TaSnRK2.64	945	103,551.67	6.31	74.21	− 0.539	nucl	PKinase
TaSnRK2.65	311	34,927.63	5.03	87.14	− 0.317	chlo	PKinase
TaSnRK3.1	486	55,169.05	8.99	84.67	− 0.547	E.R	PKinase & NAF
TaSnRK3.2	481	54,565.54	9.12	85.76	− 0.518	chlo	PKinase & NAF
TaSnRK3.3	491	55,735.96	9.12	87.98	− 0.485	chlo	PKinase & NAF
TaSnRK3.4	863	97,559.7	5.95	90.71	− 0.316	cyto	PKinase & NAF
TaSnRK3.5	507	57,577.94	8.75	84.83	− 0.545	chlo	PKinase & NAF
TaSnRK3.6	507	57,470.74	8.75	84.06	− 0.541	cyto	PKinase & NAF
TaSnRK3.7	863	97,559.7	5.95	90.71	− 0.316	chlo	PKinase & NAF
TaSnRK3.8	472	53,716.95	9.45	85.4	− 0.476	chlo	PKinase & NAF
TaSnRK3.9	472	53,612.71	9.35	84.15	− 0.488	chlo	PKinase & NAF
TaSnRK3.10	472	53,730.79	9.39	84.98	− 0.492	nucl	PKinase & NAF
TaSnRK3.11	449	51,035.83	9.19	86.84	− 0.416	chlo	PKinase & NAF
TaSnRK3.12	362	40,942.14	8.94	87.54	− 0.436	chlo	PKinase & NAF
TaSnRK3.13	444	50,281.78	9.03	86.28	− 0.429	chlo	PKinase & NAF
TaSnRK3.14	433	48,481.11	9.38	88.71	− 0.359	chlo	PKinase & NAF
TaSnRK3.15	438	49,256.87	9.25	86.78	− 0.387	chlo	PKinase & NAF
TaSnRK3.16	438	49,157.79	9.29	88.33	− 0.378	chlo	PKinase & NAF
TaSnRK3.17	356	40,111.51	8.94	86.52	− 0.345	cyto	PKinase & NAF
TaSnRK3.18	452	51,009.15	9.13	83.47	− 0.391	nucl	PKinase & NAF
TaSnRK3.19	341	38,664.29	5.45	89.97	− 0.208	cyto	PKinase & NAF
TaSnRK3.20	438	49,952.76	9.1	87.72	− 0.356	cyto	PKinase & NAF
TaSnRK3.21	466	52,443.42	9.25	82.23	− 0.445	chlo	PKinase & NAF
TaSnRK3.22	464	52,235.09	9.34	81.96	− 0.476	chlo	PKinase & NAF
TaSnRK3.23	464	52,165.04	9.34	82.18	− 0.473	chlo	PKinase & NAF
TaSnRK3.24	447	48,011.08	9.08	88.86	− 0.143	plas	PKinase & NAF
TaSnRK3.25	447	48,011.08	9.08	88.86	− 0.143	plas	PKinase & NAF
TaSnRK3.26	390	41,928.22	8.73	90.62	− 0.084	plas	PKinase & NAF
TaSnRK3.27	439	47,836.11	8.94	92.64	− 0.157	cyto	PKinase & NAF
TaSnRK3.28	439	47,867.17	9.12	92.44	− 0.156	cyto	PKinase & NAF
TaSnRK3.29	439	47,879.24	9.08	92.21	− 0.151	plas	PKinase & NAF
TaSnRK3.30	446	47,919.07	9.18	89.51	− 0.142	chlo	PKinase & NAF
TaSnRK3.31	447	49,504.86	9.09	85.55	− 0.193	chlo	PKinase & NAF
TaSnRK3.32	447	49,471.79	9	85.32	− 0.195	chlo	PKinase & NAF
TaSnRK3.33	432	47,928.07	6.41	92.11	− 0.167	cyto	PKinase & NAF
TaSnRK3.34	432	48,069.22	7.68	88.03	− 0.216	cyto	PKinase & NAF
TaSnRK3.35	433	47,848	8.46	93.23	− 0.173	cyto	PKinase & NAF
TaSnRK3.36	317	35,509.72	5.34	89.81	− 0.162	cyto	PKinase & NAF
TaSnRK3.37	433	47,892.11	8.46	92.1	− 0.156	cyto	PKinase & NAF
TaSnRK3.38	433	47,020.58	6.72	87.69	− 0.133	chlo	PKinase & NAF
TaSnRK3.39	436	47,362.85	6.48	87.32	− 0.171	chlo	PKinase & NAF
TaSnRK3.40	519	56,926.68	8.06	76.18	− 0.31	mito	PKinase & NAF
TaSnRK3.41	520	56,965.71	8.38	76.6	− 0.303	chlo	PKinase & NAF
TaSnRK3.42	522	57,208.9	8.11	76.69	− 0.316	chlo	PKinase & NAF
TaSnRK3.43	456	50,799.38	8.61	85.13	− 0.311	cyto	PKinase & NAF
TaSnRK3.44	555	61,507.58	8.96	82.43	− 0.323	chlo	PKinase & NAF
TaSnRK3.45	521	57,993.2	8.39	76.97	− 0.429	chlo	PKinase & NAF
TaSnRK3.46	519	57,926.21	8.35	77.63	− 0.417	chlo	PKinase & NAF
TaSnRK3.47	522	58,085.74	8.65	82.36	− 0.288	chlo	PKinase & NAF
TaSnRK3.48	525	58,523.38	8.77	82.8	− 0.299	chlo	PKinase & NAF
TaSnRK3.49	353	39,277.96	5.27	77.62	− 0.319	chlo	PKinase & NAF
TaSnRK3.50	462	53,394.1	8.27	72.23	− 0.704	chlo	PKinase & NAF
TaSnRK3.51	477	53,260.86	9.43	80.57	− 0.388	chlo	PKinase & NAF
TaSnRK3.52	477	53,276.92	9.41	82.2	− 0.371	chlo	PKinase & NAF
TaSnRK3.53	431	47,128.62	8.82	96.68	− 0.08	chlo	PKinase & NAF
TaSnRK3.54	431	47,227.75	8.81	96.45	− 0.099	chlo	PKinase & NAF
TaSnRK3.55	431	47,146.69	8.66	96.91	− 0.071	chlo	PKinase & NAF
TaSnRK3.56	432	47,695.96	9.13	92.31	− 0.204	mito	PKinase & NAF
TaSnRK3.57	432	47,647.9	8.68	92.31	− 0.177	mito	PKinase & NAF
TaSnRK3.58	432	47,653.86	9.19	92.52	− 0.196	mito	PKinase & NAF
TaSnRK3.59	433	47,621.03	9.19	91.66	− 0.187	mito	PKinase & NAF
TaSnRK3.60	433	47,516.97	9.28	92.33	− 0.166	cyto	PKinase & NAF
TaSnRK3.61	432	47,421.87	9.51	90.51	− 0.185	mito	PKinase & NAF
TaSnRK3.62	449	50,551.69	6.59	89.02	− 0.337	cyto	PKinase & NAF
TaSnRK3.63	449	50,513.77	7.67	90.33	− 0.33	cyto	PKinase & NAF
TaSnRK3.64	370	41,272.64	9.38	91.16	− 0.209	nucl	PKinase & NAF
TaSnRK3.65	402	45,089.86	8.9	88.78	− 0.264	nucl	PKinase & NAF
TaSnRK3.66	446	50,054.68	8.87	90.07	− 0.214	cyto	PKinase & NAF
TaSnRK3.67	446	49,921.74	8.78	92.04	− 0.18	cyto	PKinase & NAF
TaSnRK3.68	462	51,797.34	9.15	82.53	− 0.429	chlo	PKinase & NAF
TaSnRK3.69	461	51,740.29	9.15	82.71	− 0.429	chlo	PKinase & NAF
TaSnRK3.70	453	50,950.38	9.16	83.31	− 0.434	chlo	PKinase & NAF
TaSnRK3.71	445	50,038.32	7.67	79.33	− 0.407	chlo	PKinase & NAF
TaSnRK3.72	443	49,798.06	7.23	80.14	− 0.388	chlo	PKinase & NAF
TaSnRK3.73	439	50,259.7	7.66	86.58	− 0.475	chlo	PKinase & NAF
TaSnRK3.74	439	50,259.7	7.66	86.58	− 0.475	chlo	PKinase & NAF
TaSnRK3.75	439	50,248.75	7.19	85.69	− 0.466	chlo	PKinase & NAF
TaSnRK3.76	447	50,443.98	8.69	86.4	− 0.371	chlo	PKinase & NAF
TaSnRK3.77	447	50,460.98	8.7	85.97	− 0.392	chlo	PKinase & NAF
TaSnRK3.78	447	50,532.98	8.6	84.88	− 0.414	chlo	PKinase & NAF
TaSnRK3.79	382	43,428.98	8.06	88.38	− 0.363	chlo	PKinase & NAF
TaSnRK3.80	443	50,260.79	8	85.21	− 0.381	chlo	PKinase & NAF
TaSnRK3.81	449	50,983.68	8.27	86.24	− 0.373	chlo	PKinase & NAF
TaSnRK3.82	439	48,837.15	8.17	98.15	− 0.181	pero	PKinase & NAF
TaSnRK3.83	446	49,653.72	6.19	96.61	− 0.224	nucl	PKinase & NAF
TaSnRK3.84	529	59,022.41	8.38	87.88	− 0.233	cyto	PKinase & NAF
TaSnRK3.85	417	47,498.45	7.57	86.02	− 0.432	cyto	PKinase & NAF
TaSnRK3.86	417	47,486.49	7.58	85.78	− 0.43	cyto	PKinase & NAF
TaSnRK3.87	451	51,041.42	6.84	85.81	− 0.416	cyto	PKinase & NAF
TaSnRK3.88	471	52,048.56	7.68	90.91	− 0.263	cyto	PKinase & NAF
TaSnRK3.89	466	51,625.04	6.95	91.89	− 0.261	chlo	PKinase & NAF
TaSnRK3.90	448	49,648.95	6.95	94.26	− 0.229	cyto	PKinase & NAF
TaSnRK3.91	465	51,705.23	6.74	91.25	− 0.277	cyto	PKinase & NAF
TaSnRK3.92	466	51,766.51	7.65	93.11	− 0.243	cyto	PKinase & NAF
TaSnRK3.93	466	51,548.11	7.22	90.82	− 0.238	cyto	PKinase & NAF
TaSnRK3.94	466	51,838.48	7.64	91.24	− 0.267	cyto	PKinase & NAF
TaSnRK3.95	480	53,041.06	6.9	94.9	− 0.159	cyto	PKinase & NAF

### Phylogenetic analysis of SnRK genes family

**Figure 1:**
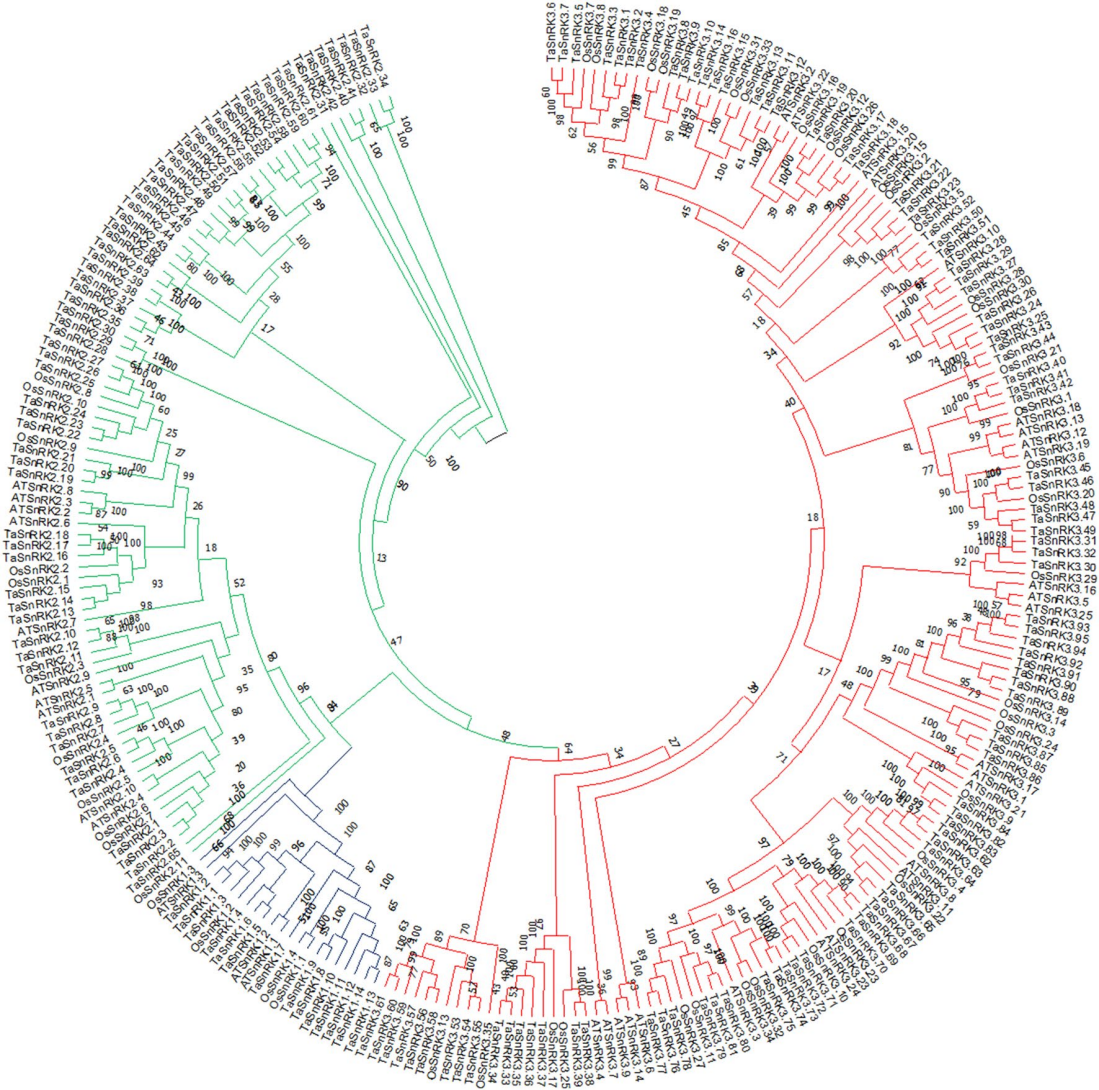
Phylogenetic tree of *SnRK* genes from *T. aestivum, A. thaliana, and O. sativa*. 174 *SnRK* genes from wheat, 38 from Arabidopsis and 50 from rice were clustered into three subgroups (SnRK1, SnRK2 and SnRK3). Wheat SnRK1, 2 and 3 subgroups are denoted as blue, green and red, respectively. The tree was generated using MEGA X software using the neighbor-joining method.

To study the evolutionary relationships among SnRK proteins and their classification, we performed unrooted phylogenetic tree analysis using the full-length amino acid sequences of 174 *SnRKs* genes of *T. aestivum*, 38 of *A. thaliana* and 50 of *O. sativa* (Fig. 1). The clustering of 38 AtSnRKs into three groups was reported earlier^3^. Based on the phylogenetic analysis and domains presence, 174 TaSnRKs were also divided into three groups in this study. Of these, 14 proteins in the TaSnRK1 subfamily have Pkinase (PF00069 of Pfam), UBA (PF00627), and KA1 (PF02149) domains, whereas 65 proteins in the TaSnRK2 subfamily have Pkinase domains with strong resemblance to AtSnRK2 subfamily, and 95 proteins in the SnRK3 subfamily have Pkinase and NAF (PF03822) domain (Fig. 1).

### Motif composition and gene structural analysis of the SnRK gene family in *T. aestivum*

**Figure 2:**
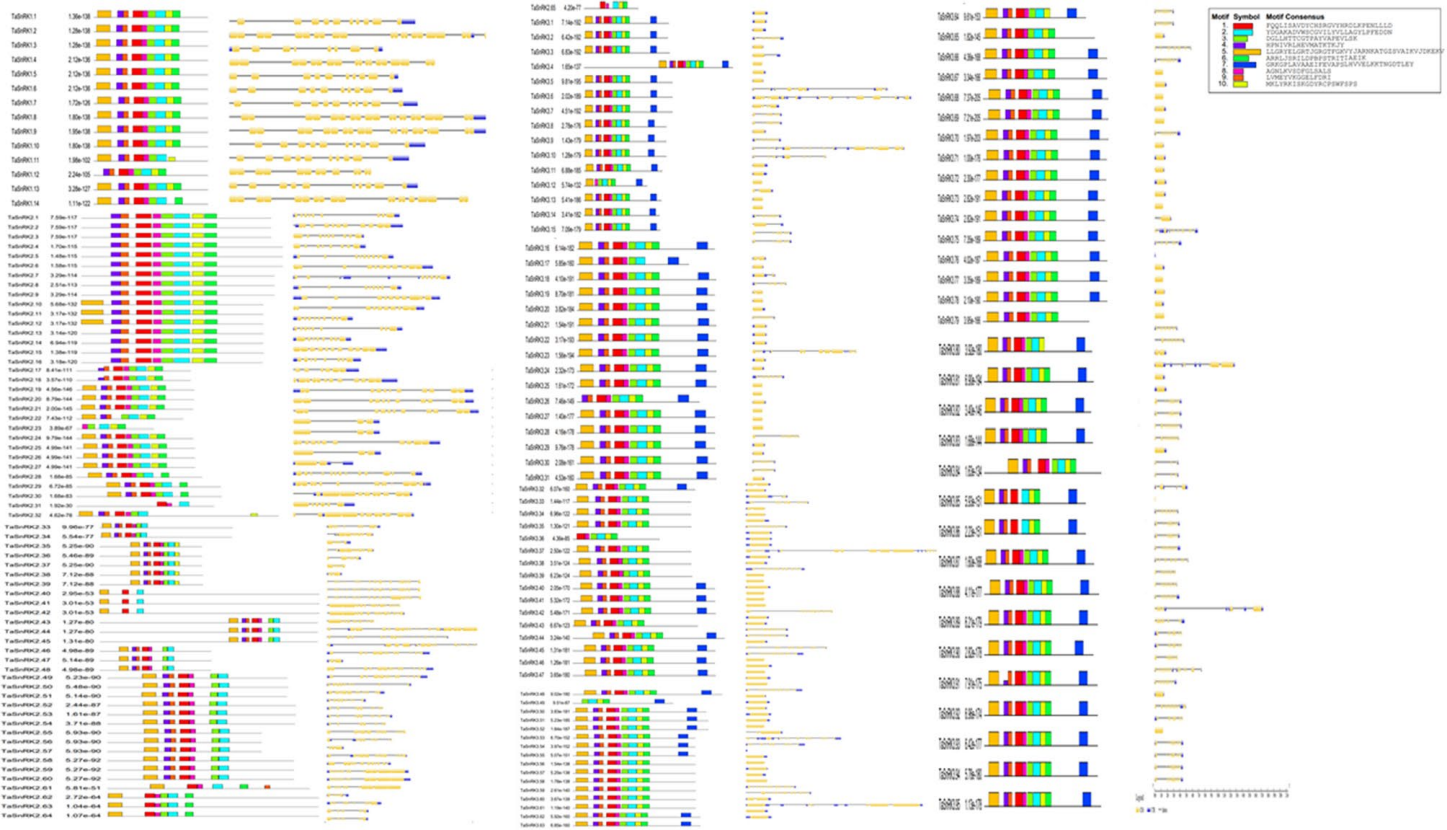
(**A**) Arrangement of ten conserved motifs in the *TaSnRK *genes following MEME analysis*. *Different colored boxes represent different motifs and their position in each sequence of *TaSnRK *genes. (**B**) Gene structure of wheat SnRK. Exons are indicated in yellow rectangles and grey line connecting two exons represent introns.

MEME analysis showed that 10 conserved motifs were identified in TaSnRK proteins (Fig. 2A). The conserved motif's sequence and length details have been listed in Supplementary Table S1. The conserved Pkinase domain including the pattern 1, 2, 3, 5, 6 was discovered in all *TaSnRK* genes in this investigation (Supplementary Fig. 1). Furthermore, *TaSnRK* genes from the same subfamily have comparable pattern compositions, but *TaSnRK* genes from different subfamilies have varied motif compositions. TaSnRK1 subfamily genes have 9 motif (motifs 1, 2, 3, 4, 5, 6, 7, 8 and 10) while TaSnRK2 subfamily genes had either motifs 1, 2, 4, 6, 8 or motifs 1, 2, 3, 4, 5 and 10. TaSnRK3 subfamily genes had 10 motifs while a few of them do not have any motif (Fig. 2A). In conclusion, the comparable gene architectures and conserved motif compositions of SnRK genes within the same subfamily substantially support the phylogenetic analysis based subfamily classifications.

The exon–intron structure of *TaSnRK* genes showed that the TaSnRK1 subfamily genes have more than 10 exons, while the TaSnRK2 subfamily genes have 2–34 exons followed by TaSnRK3 subfamily which have 1–33 exons (Fig. 2B). Notably, 14 genes are intron-less. In addition, the TaSnRK3 family is divided into two subgroups. The genes in SnRK3 subgroup 1 had more than 10 exons, while the genes in subgroup 2 had less than four exons.

### Analysis of chromosomal location and orthologous genes in *T. aestivum*

**Figure 3:**
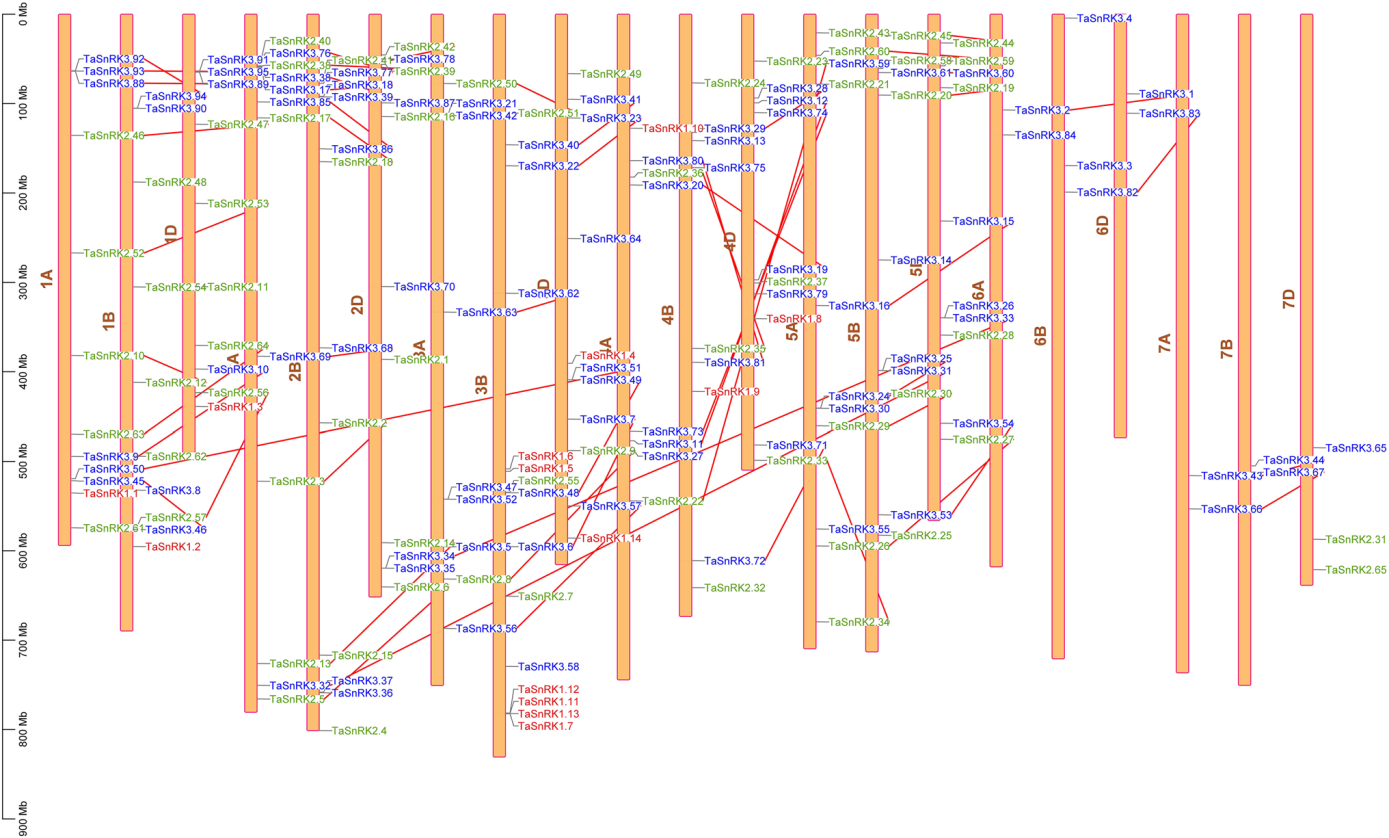
Distribution of *TaSnRK* genes on the 21 chromosomes of wheat and within the three sub-genomes. Black lines representing the gene pairs. Physical map showing the chromosomal distribution, with position on the left side scale bar and the name of the gene on right side.

The chromosomal distribution of all *TaSnRK* genes across the genome was investigated which provides useful information on the genomic regions (Fig. 3). The A sub-genome had 56 *TaSnRK* genes, comprising 2 genes from TaSnRK1 subfamily, 21 genes from TaSnRK2 subfamily, and 33 genes from TaSnRK3 subfamily, whereas the B sub-genome had 61 genes, comprising 8 genes from TaSnRK1 subfamily, 21 of TaSnRK2 subfamily, and 32 of TaSnRK3 subfamily. However, 4 genes of TaSnRK1 subfamily, 23 genes of TaSnRK2 subfamily, and 30 genes of TaSnRK3 subfamily were found on the D sub-genome (Supplementary Fig. S2A). These findings suggested that TaSnRK genes were distributed randomly throughout the A, B, and D chromosomes (Supplementary Fig. S2B).

In this study, 57 orthologous pairings were identified between *T. aestivum* and *H. valgare*, while 166 between *T. aestivum* and *A. thaliana*. However, 53 orthologous genes were found within wheat species for instance between *T. aestivum* and *T. urartu*, while 102 orthologous genes between *T. aestivum* and *Ae. dicoccoides* and 63 between *T. aestivum* and *Ae. tauschii* (Supplementary Table S3).

We identified 11 segmental events across different chromosomes and 2 tandom duplication occurrences in the same chromosome using the BLAST and MCScanX techniques. The findings revealed that gene duplication may have produced some *TaSnRK* genes, and that segmental duplication events were important in the growth of *TaSnRK* genes in the wheat genome. We also looked into the frequency of tandem duplication occurrences. There were 45 *TaSnRK* gene pairs found in this area all of which were strongly related. (Supplementary Table S4). However, the identities of these were > 80%, indicating that they were included into tandem duplication occurrences.

**Figure 4:**
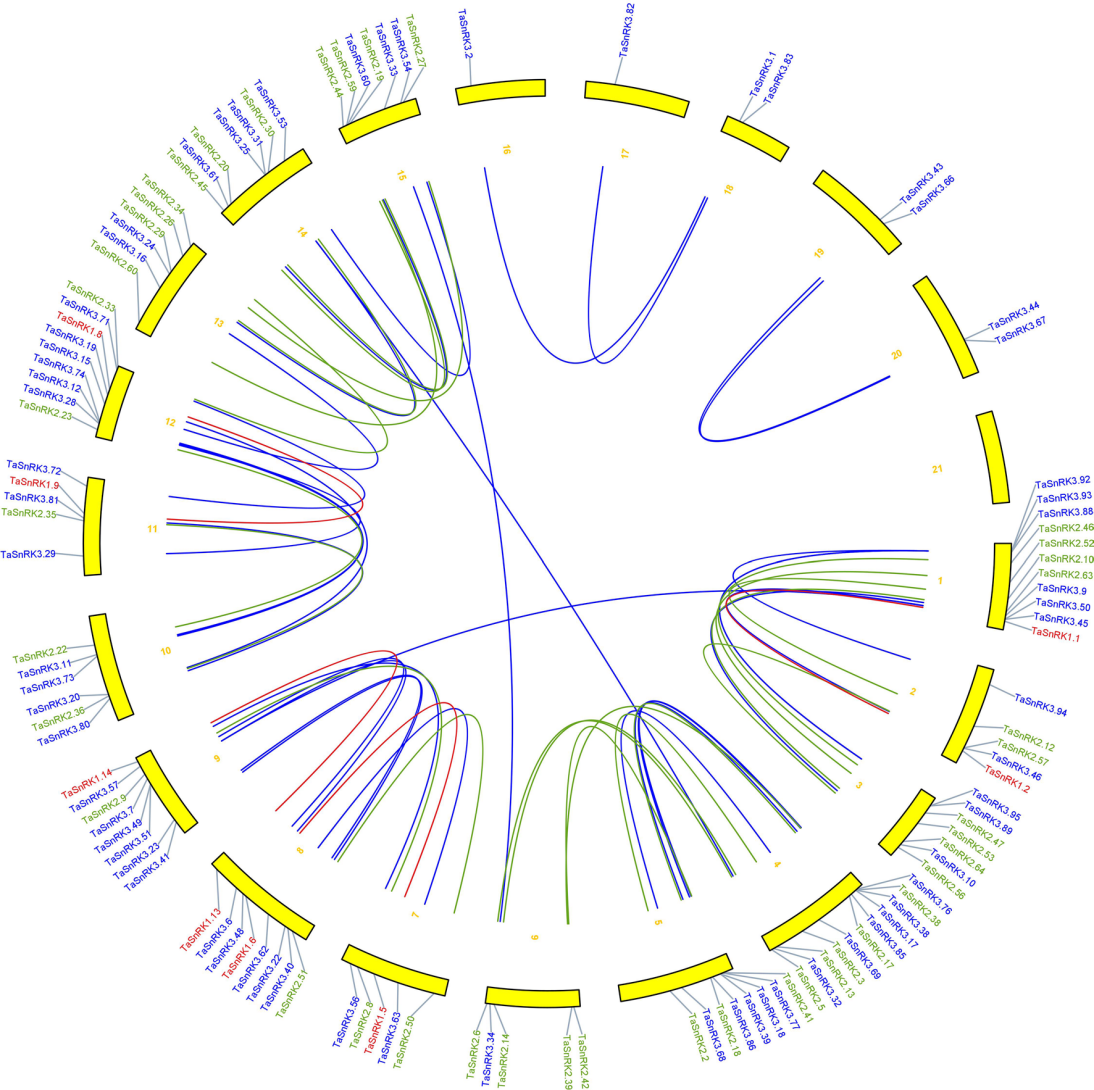
The synteny analysis of TaSnRK family in *T. aestivum.* Different colours represent SnRK subfamilies red lines indicate duplicated TaSnRK1 subfamily gene pairs, green lines indicated TaSnRK2 subfamily gene pairs and blue represented TaSnRK3 subfamily. The chromosome number is indicated at the bottom of each chromosom.

We looked at the duplication events of the *TaSnRK* gene in the wheat genome since gene duplication has a big impact on the emergence of new functionalities and gene families. In addition, 55 gene pairs were found to be duplicated as shown in Fig. 4.

**Figure 5:**
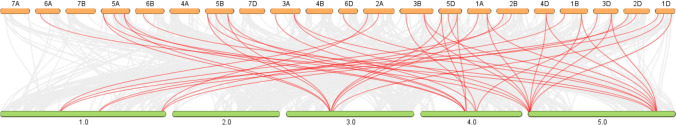
Synteny analysis of SnRK genes between *Triticum aestivum* (Orange colour) and *Arabidopsis thaliana *(green colour). Gray lines in the background indicate the collinear blocks within *T. aestivum* and *A. thaliana*, while the red lines highlight the syntenic SnRK gene pairs.

Furthermore, the synteny of *SnRK* gene pairs between *T. aestivum* genome and *A. thaliana* genome was performed. The result showed that 166 *SnRK* genes of *T. aestivum* exhibiting syntenic relationship with *AtSnRK* genes (Fig. 5 and Supplementary Table S3), suggesting that these genes might have contributed to the evolution of *TaSnRK* gene family. K_s_ values, K_a_ values, K_a_/K_s_ ratios and divergence time of paralogous and orthologous on SnRK family genes were estimated to assess the evolutionary constraints undertaking (Supplementary Table S4). The K_a_/K_s_ ratios of the majority of segmentally duplicated *TaSnRK* gene pairs were < 1, the mean values of TaSnRK3 gene pairs (K_a_/K_s_ = 0.30) and TaSnRK2 (K_a_/K_s_ = 0.35) were lower than TaSnRK1 (K_a_/K_s_ = 0.41). Furthermore, segmental gene divergence time spans from 18.76 to 34.97 Mya. These findings showed that the *TaSnRK* gene family may have been subjected to significant purifying selection during evolution.

### Promoter analysis

PlantCARE was used to look at *cis*-elements (1.5 kb upstream from ATG) in order to better understand the function and regulatory processes of *TaSnRK* genes. We found 129 out of 174 *TaSnRK* genes had *cis*-elements (Supplementary Fig. S3, Supplementary Table S5). MyB, ABRE, and LTRE *cis*-elements were found to be involved in drought, ABA, and low-temperature responses. Auxin (9.77%), MeJA (51.72%), and Gibberallin (11.49%) *cis*-elements were found in phytohormones. It was also shown that most genes have many *cis*-element types. In addition, the TaSnRK1 (30.50) family had more *cis*-elements than the TaSnRK2 (19.29) or TaSnRK3 (23.81) families (Supplementary Table S5). Finally, the *cis*-elements study revealed that most *TaSnRK* genes can respond to a variety of environmental challenges, and that distinct subfamily genes can be regulated in various ways.

### MicroRNAs targeting TaSnRK

The role of miRNAs in controlling the expression of *TaSnRK* genes have been investigated using the psRNATarget server. We predicted 16 *TaSnRK* genes as possible targets for eight different miRNAs (Table 2). Tae-miR319 implicated in the regulation of seven *TaSnRK* genes (TaSnRK3.33, TaSnrK3.36, TaSnRK2.37, TaSnRK2.35, TaSnRK2.36, TaSnRK3.38, TaSnRK2.39). Tae-miR408 accounted for regulating expression of three *TaSnRK* genes (TaSnRK3.63, TaSnRK3.64, TaSnRK3.62). The expression of *TaSnRK* genes (TaSnRK1.3, TaSnRK1.2, and TaSnRK2.39, TaSnRK2.38) may be influenced by the Tae-miR164 and Tae-miR167b (Table 2). Tae-miR1119 and Tae-miR160 were predicted to regulate the expression of TaSnRK3.38 and TaSnRK3.74, respectively.

**Table 2:** Prediction Tae-MIR genes and their targets by using the psRNATarget server with default parameters.

miRNA_Acc	Target_Acc	Target_start	Target_end	miRNA_aligned_fragment	Target_aligned_fragment	Inhibition
tae-miR167b	TaSnRK2.39	233	253	UGAAGCUGACAGCAUGAUCUA	AUGAUCCUGCGGUCAUCUUCA	Translation
	TaSnRK2.38	1610	1630	UGAAGCUGACAGCAUGAUCUA	AUGAUCCUGCGGUCAUCUUCA	Translation
tae-miR408	TaSnRK2.65	876	896	CUGCACUGCCUCUUCCCUGGC	AGCACCGAGGAGGCAGAGCAG	Cleavage
tae-miR1119	TaSnRK3.38	50	73	UGGCACGGCGUGAUGCUGAGUCAG	AGUGAGCAGCAGCGCGUCGUGUUA	Cleavage
tae-miR160	TaSnRK3.74	332	352	UGCCUGGCUCCCUGUAUGCCA	AGGCAGGCAGGCAGGCAGGCA	Translation
tae-miR319	TaSnRK3.33	905	925	UUGGACUGAAGGGAGCUCCCU	AUGGAGCACCCUUGGGUACAA	Cleavage
	TaSnRK3.36	898	918	UUGGACUGAAGGGAGCUCCCU	AUGGAGCACCCUUGGGUACAA	Cleavage
	TaSnRK2.38	2022	2042	UUGGACUGAAGGGAGCUCCCU	GAGGUACUCCUUUCAGACCAA	Cleavage
	TaSnRK2.39	645	665	UUGGACUGAAGGGAGCUCCCU	GAGGUACUCCUUUCAGACCAA	Cleavage
	TaSnRK2.37	1269	1289	UUGGACUGAAGGGAGCUCCCU	GAGGUACUCCUUUCAGACUAA	Cleavage
	TaSnRK2.35	1219	1239	UUGGACUGAAGGGAGCUCCCU	GAGGUACUCCUUUCAGACUAA	Cleavage
	TaSnRK2.36	1031	1051	UUGGACUGAAGGGAGCUCCCU	GCGGUACUCCUUUCAGACUAA	Cleavage
tae-miR164	TaSnRK1.3	1677	1697	UGGAGAAGCAGGGCACGUGCA	GACCUGUGUUCUGCCUUUCUA	Cleavage
	TaSnRK1.2	1579	1599	UGGAGAAGCAGGGCACGUGCA	GACCUGUGUUCUGCCUUUCUA	Cleavage
tae-miR408	TaSnRK3.63	184	204	CUGCACUGCCUCUUCCCUGGC	GGAGGGGGAGGGGCGCUGCGG	Cleavage
	TaSnRK3.64	101	121	CUGCACUGCCUCUUCCCUGGC	GGAGGGGGAGGGGCGCUGCGG	Cleavage
	TaSnRK3.62	120	140	CUGCACUGCCUCUUCCCUGGC	GGAGGGGGAGGGGCGCUGCGG	Cleavage

### Functional annotation of Hub genes and interacting network analysis

**Figure 6:**
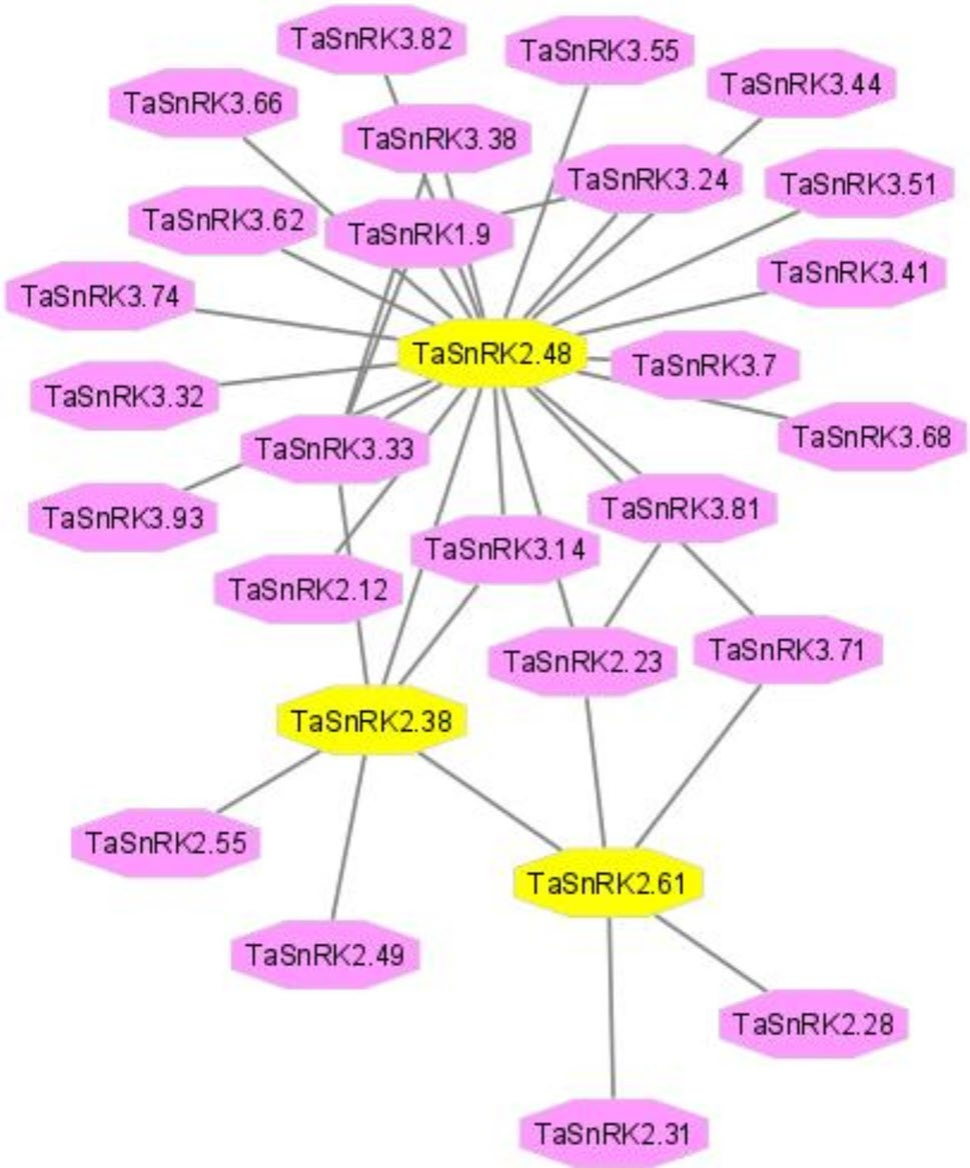
The predicted protein–protein interactions network of SnRK in wheat based on the *Arabidopsis* orthologs using Cytoscape.

Based on their involvement in a biological and cellular process, we studied the function of potential hub genes. String database contains a collection of predicted and experimentally confirmed protein–protein interactions in wheat and other species. However, wheat SnRK found in string database is linked to them as well as numerous metabolic and regulatory processes. SnRK were defined based on the interaction observed.* TaSnRK2.48* is included in the first, which serves as the network’s (Fig. 6). Here, the cluster is directly linked to TaSnRK3 subfamily (*TaSnRK3.7, TaSnRK 3.14, TaSnRK 3.24, TaSnRK3.32, TaSnRK3.33, TaSnRK3.38, TaSnRK3.41, TaSnRK3.44, TaSnRK3.55, TaSnRK3.62 TaSnRK3.71, TaSnRK3.74, TaSnRK3.81, TaSnRK3.82, TaSnRK3.84 and TaSnRK3.93*). These genes involved in Ca-dependent processes like as autophagy and maintenance. The second interaction with SnRK 2 subfamily (*TaSnRK2.12*, *TaSnRK2.27* and *TaSnRK2.36*) was primarily linked to OST. Whereas the primary role of OST is to act as an activator of the abscisic acid (ABA) signalling pathway, which governs several ABA responses including stomata closure in response to dehydration, plant diseases, or changes in atmospheric relative humidity (RH). Pathogen-associated molecular patterns (PAMPs) (e.g. flg22 and LPS) of pathogenic bacteria such as *P.syringae* pv. tomato (Pst) and *E. coli* O157:H7 are required for stomata closer. *TaSnRK 2.28* and *TaSnRK1.9* have a direct connection that plays a crucial function in fatty acid synthesis. Further, there is no direct evidence of a direct interaction between TaSnRK2.61 and TaSnRK2.48 (Fig. 6). Apart from that, *TaSnRK2.61* is the network’s second hub gene, which mainly interacts with SnRK2 subfamily, such as (*TaSnRK2.16*, *TaSnRK2.28*, *TaSnRK2.31*, *TaSnRK2.38* and *TaSnRK3.71*), which are expressed preferentially in guard cells and appear to be involved ABA signalling mediating by reactive oxygen species.

### Expression profile of SnRK genes in different tissues under abiotic stress

To understand more about how *TaSnRK*s are involved in development and stress responses, we utilized the expVIP database to retrieve TPM values for all TaSnRKs from experiments including abiotic stress and various developmental stages. These TPM data were used to generate Heatmaps. The expression patterns of 174 *TaSnRK* genes in various wheat tissues were evaluated.

**Figure 7:**
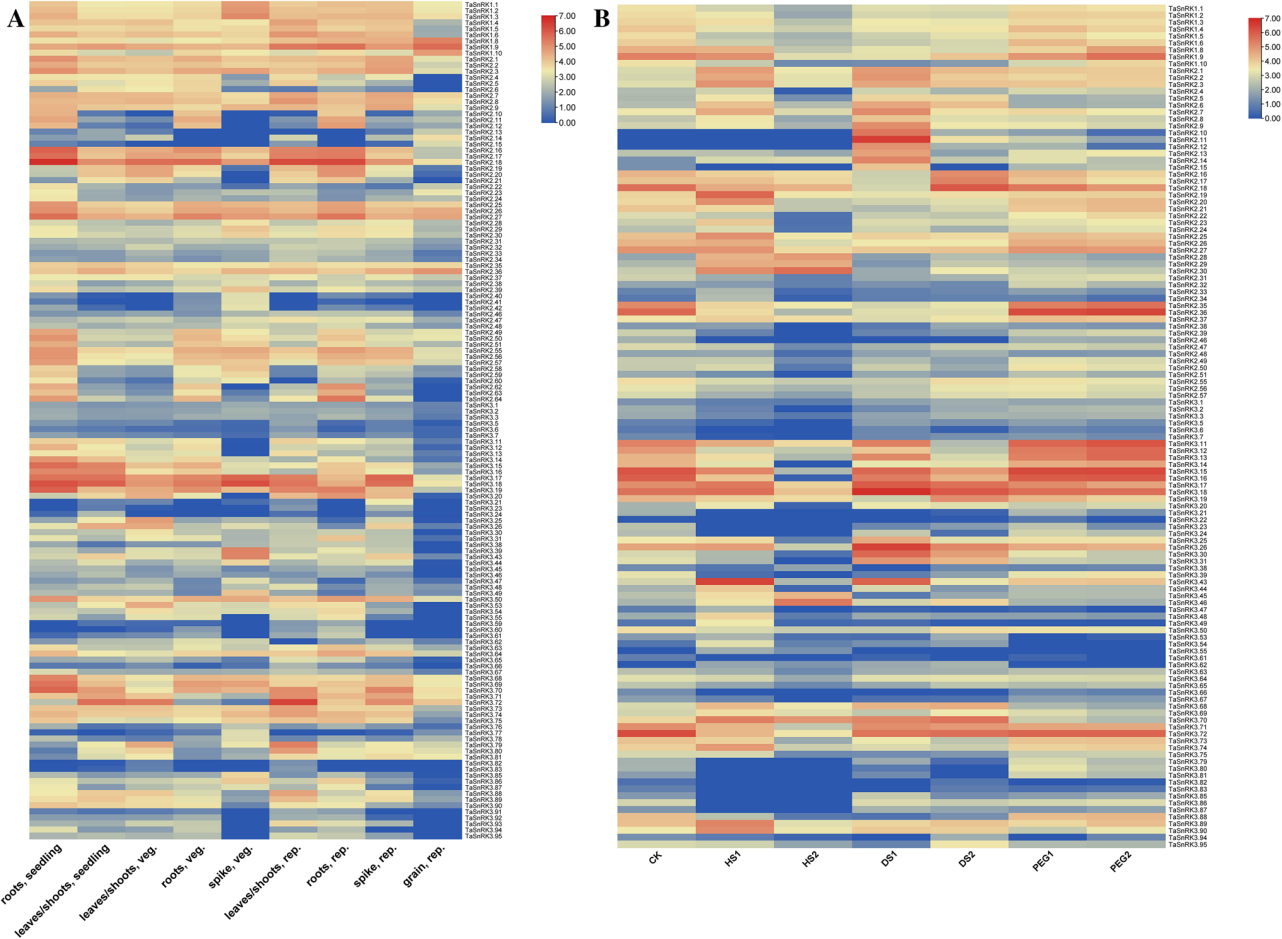
Heatmap of *TaSnRK* genes (**A**) tissue specific expression in leaf, root, shoot and spike at seedling, vegetative and reproductive stages (**B**) abiotic stress specific expression under drought, heat and osmotic stress at seedling stage. The color scale represents log_2_ expression values. Ck-control; HS1-heat stress at 1hr; HS2-heat stress at 6hr; DS1- drought stress at 1hr; DS2- drought stress at 6hr; PEG1- osmotic stress at 2hr; PGE2- osmotic stress at 12 hr.

Five different tissues at three different developmental stages were taken for this study. The time points are the Zadoks scale (Z-scale). Different TaSnRK sub-families show differential induction in different tissues (Fig. 7A). TaSnRKs such as *TaSnRK1.1, TaSnRK1.2, TaSnRK1.3, TaSnRK1.4, TaSnRK1.5, TaSnRK1.6, TaSnRK1.8, TaSnRK1.9 and TaSnRK1.10, TaSnRK2.1, TaSnRK2.2, TaSnRK2.3, TaSnRK2.4, TaSnRK2.5, TaSnRK2.6, TaSnRK2.7, TaSnRK2.8, TaSnRK2.9, TaSnRK2.10, TaSnRK2.11, TaSnRK2.12, TaSnRK2.16, TaSnRK2.17, TaSnRK2.18, TaSnRK2.25, TaSnRK2.26, TaSnRK2.27, TaSnRK2.50, TaSnRK2.51, TaSnRK2.55, TaSnRK2.56, TaSnRK2.57, TaSnRK2.62, TaSnRK2.64, TaSnRK3.16, TaSnRK3.17, TaSnRK3.18, TaSnRK3.19, TaSnRK3.30, TaSnRK3.31, TaSnRK3.43, TaSnRK3.44, TaSnRK3.50, TaSnRK3.53, TaSnRK3.54, TaSnRK3.55, TaSnRK3.68, TaSnRK3.69, TaSnRK3.70, TaSnRK3.71, TaSnRK3.72, TaSnRK3.86, TaSnRK3.87, TaSnRK3.89, TaSnRK3.90* and *TaSnRK3.9*5 showed induction in spike tissues at Z39 stage. Whereas, *TaSnRK1.1, TaSnRK1.2, TaSnRK1.3, TaSnRK1.4, TaSnRK1.5, TaSnRK1.6, TaSnRK1.8, TaSnRK1.9* and *TaSnRK1.10* etc. were up regulated in leaves tissues at Z75 stage. Multiple TaSnRKs were induced at different spike stages especially at Z39 stage. Forty genes of TaSnRK3 subfamily were induced in root tissues at Z10 stage. This suggests different TaSnRKs might be involved in development of different tissues at different stages. Many TaSnRKs showed induction at the dough development stage in wheat such as *TaSnRK1.1, TaSnRK1.2, TaSnRK1.3, TaSnRK1.8 to, TaSnRK1.9 and TaSnRK1.10, TaSnRK2.1, TaSnRK2.2, TaSnRK2.3, TaSnRK2.7, TaSnRK2.8, TaSnRK2.9, TaSnRK2.10, TaSnRK2.11, TaSnRK2.12, TaSnRK2.14, TaSnRK2.15, TaSnRK2.16, TaSnRK2.17, TaSnRK2.18, TaSnRK2.25, TaSnRK2.26, TaSnRK2.27, TaSnRK2.35, TaSnRK2.36, TaSnRK2.37, TaSnRK2.38, TaSnRK2.39, TaSnRK2.49, TaSnRK2.50, TaSnRK2.51, TaSnRK2.55, TaSnRK2.56*, and *TaSnRK2.57*. However, the expression of TaSnRK3 subfamily was down regulated in Z85 stage as the grain matures (Fig. 7A). Very few members of the TaSnRKs sub-family showed no induction during any of the different developmental stages e.g., *TaSnRK1.7, TaSnRK1.11, TaSnRK1.12, TaSnRK1.13, TaSnRK1.14, TaSnRK2.43, TaSnRK2.44, TaSnRK2.45, TaSnRK2.46, TaSnRK3.37, TaSnRK3.40, TaSnRK3.42, TaSnRK3.51, TaSnRK3.82*, and *TaSnRK3.83*. The SnRK subfamily 1, 2 and 3 were induced in leaf tissue at Z10 stages.

The expression patterns of *TaSnRK* family genes in wheat tissues under abiotic stresses were evaluated (Fig. 7B). A total of 16 genes with high expression levels in all studied tissues under abiotic stresses (log_2_-based values > 5) were assigned to sub-group1. *SnRK3.40*, for example, was shown to be highly expressed in all vegetative organs, with log_2_-based average values. In sub-group2, the expression levels of 55 *TaSnRK* genes were significantly lower across all detected tissues (log_2_-based values > 2). In sub-group3, 103 *TaSnRK* genes were involved with the lowest expression levels in diverse tissues at various stages. Meanwhile, sub-group1 had one TaSnRK1, 6 TaSnRK2 genes and 9 TaSnRK3 genes; Sub-group2 had 8 TaSnRK1, 21 TaSnRK2, and 26 TaSnRK3 genes and Sub-group3 had 5 TaSnRK1, 38 TaSnRK2, and 69 TaSnRK3 genes (Fig. 7A). The expression pattern of *TaSnRK* genes was nonetheless studied using drought, salinity and heat stresses^26^. The expression levels of *TaSnRK* genes have been significantly changed under various abiotic stresses (Fig. 7B). *TaSnRK3.17* and *TaSnRK3.18*, for example, were highly stimulated by all treatments, whereas *TaSnRK3.15* and *TaSnRK3.16* responded to dehydration by increasing their levels of expression. But, *TaSnRK 3.37*, *TaSnRK3.45*, and *TaSnRK3.46* have demonstrated high expression levels in response to heat and drought stress (Fig. 7B). These results demonstrated that *TaSnRK*s have a large variety of patterns of expression and that even genes within the same subfamily had different patterns of expression.

### Validation of TaSnRK for abiotic stresses using qRT-PCR

**Figure 8:**
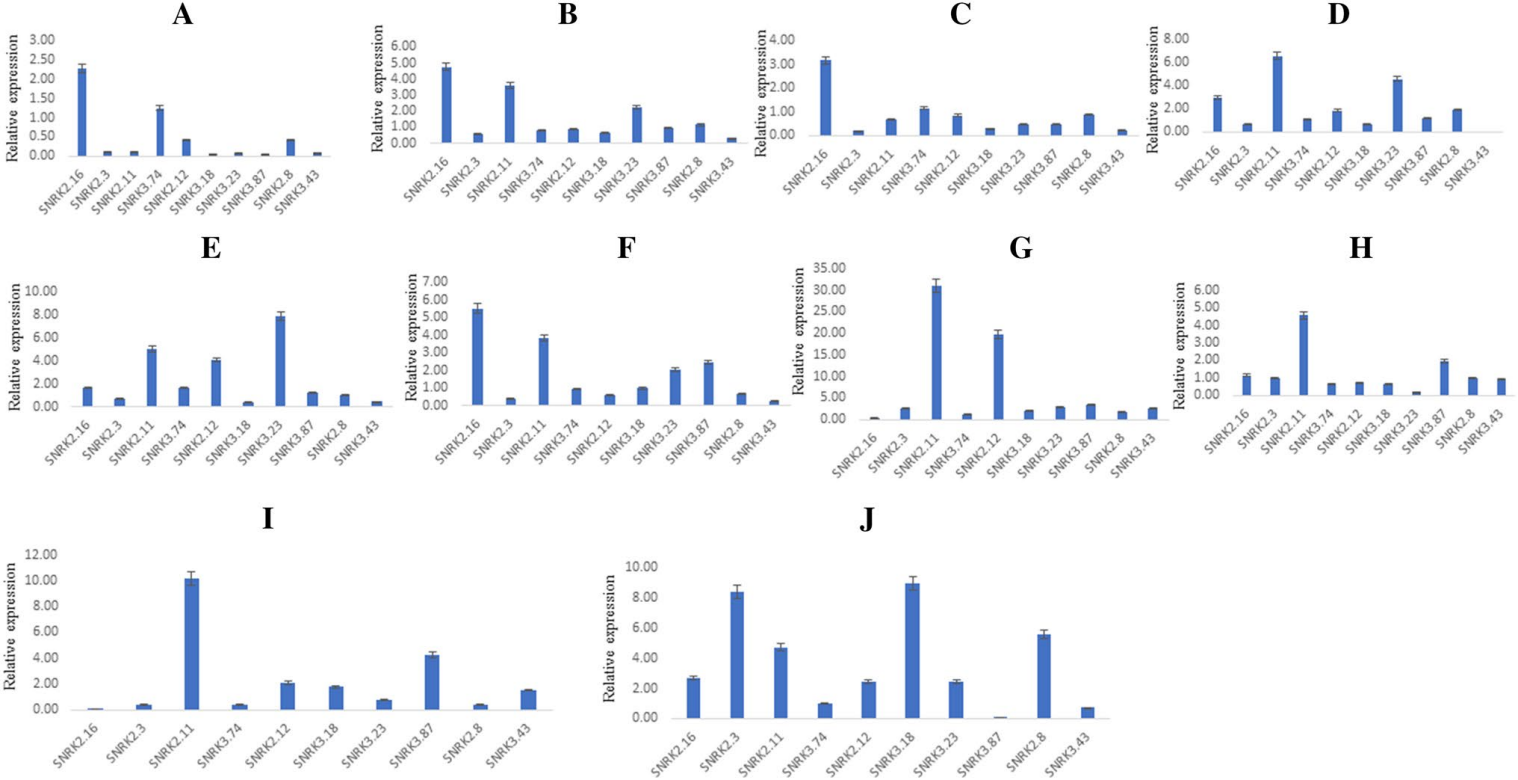
qRT-PCR based differential expression analysis of 10 *TaSnRK* genes under abiotic stress. Heat stress treatment at 37 °C (**A**) HS240, & (**B**) RAJ3765; Heat stress treatment at 42 °C (**C**) HS240, & (**D**) RAJ3765; Salt stress treatment at 150 mM NaCl (**E**) Kharchia65, (**F**) HD2687 at 12 h and (**G**) Kharchia 65, (**H**) HD 2687 for 24 h; drought stress at 25% (v/v) PEG treatment (**I**) C306 and (**J**) Wl711 for 24 h. The error bars indicate the standard deviation.

Members of the SnRK family serve critical roles in plant abiotic stress response. In plants, however, the specific mechanism underpinning SnRK function is not fully understood. The expression analysis of SnRK2s and SnRK3s in leaf tissue was identified by qRT-PCR following different stress treatments at different time intervals. This was to study the function of TaSnRK2 and TaSnRK3 genes in responding to salt, heat, and drought stress (Fig. 8).

**Figure 9:**
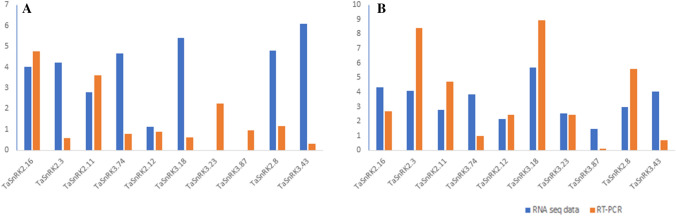
Comparison between magnitude of gene expression of RNA-seq and qRT-PCR data of heat stress (**A**) and drought stress (**B**).

*TaSnRK2.16* expression was fivefold higher in HS240 after a 37 °C heat stress treatment (Fig. 8A), followed by *TaSnRK3.74* (~ twofold) and TaSnRK2.16 (~ three fold) after a 42 °C heat stress (Fig. 8C). At 37 °C, the expression levels of four genes, *TaSnRK2.16, TaSnRK2.11, TaSnRK3.23*, and *TaSnRK2.8* were up-regulated (Fig. 8B) compared to 42 °C heat stressed Raj3765 genotype (Fig. 8D). Furthermore, expressions of *SnRK* genes were carried out at different time intervals to investigate the role of SnRK during salt stress treatment. Three genes, *TaSnRK2.11, TaSnRK2.12*, and *TaSnRK3.23* had up to eightfold higher expression levels in Kharchia65 (Fig. 8E) whereas four *SnRK* gens in HD2687 had upto 5.5-fold higher expression levels at 12 h (Fig. 8G). While at 24 h after salt stress, expression levels of two genes, *TaSnRK2.11* and *TaSnRK2.12* rose 32- and 20-folds in wheat genotype Kharchia 65 (Fig. 8F) respectively, followed by *TaSnRK2.11* (five fold) in HD2687 (Fig. 8H) at. We chose two genotypes i.e. C306 and WL711, to test under drought stress. In wheat genotype C306, *TaSnRK2.11* expression was tenfold up-regulated, but TaSnRK3.87 expression was fourfold up-regulated (Fig. 8I), whereas *TaSnRK2.3*, *TaSnRK2.11*, *TaSnRK3.18,* and *TaSnRK2.8* was more than fourfolds (Fig. 8J) in WL711. Abiotic tolerance in wheat might be improved by utilizing a higher expression level of TaSnRK2.11 which has been shown to increase under salinity, drought, and heat stress conditions. We analysed the RNA-seq and qRT-PCR data of SnRK genes to determine the magnitude of RNA transcription level (Fig. 9). A total of ten transcripts were chosen for this. The results revealed that the log fold-change values of these selected transcripts were found comparable in correspondence with RNA-seq results (Fig. 9A,B).

## Discussion

This study identified 174 *TaSnRK* genes in the *T. aestivum* genome, which were classified as TaSnRK1, TaSnRK2, or TaSnRK 3 based on their subfamily classification. The *TaSnRK* gene family was carefully searched, including evolutionary linkages, protein patterns, gene architectures, gene duplication, distributions of chromosomal, and the promoter *cis*-elements. This research progresses towards the future functionality of *SnRK* genes in order to improve abiotic stress adaption of plant. *SnRK* genes were already reported in *A. thaliana*^4^, *O. sativa*^27^, *B. distachyon*^28^, and *E. grandis*^29^ having 39, 48, 44, and 34, respectively. The number of *TaSnRK* genes in the genome of *T. aestivum* is substantially higher than in diploid plants. There were 14 TaSnRK1 genes, 65 TaSnRK2 genes , and 95 TaSnRK3 genes were discovered and classified into three subfamilies. According to more detailed description, *T. aestivum* and other species have similar member proportions for each subfamily. *T. aestivum* is a naturally occurring amphidiploid evolved from *T. urartu* (AA), *A. speltoides* (BB), and *A. tauschii* (DD). There were 56, 61, and 57 *TaSnRK* genes discovered in the A, B, and D sub-genomes, respectively demonstrating that *SnRK* genes had a similar functional role in progenitor species.

Although the conserved domains of the SnRK subfamily genes differ, the N-terminal protein kinase domain is retained. It has been discovered that SnRK3 subfamily genes interact with CBLs in a calcium-dependent manner due to the NAF domain. Furthermore, the NAF domain identifies a set of heterologous kinases that CBL calcium sensor protein targets and participate in a range of signalling cascades^8^. According to this study, distinct TaSnRKs subfamily genes shared different types of conserved domains. This might imply that the TaSnRK genes family is functionally diverse based on the domains contained.

In the *AtSnRK* and *TaSnRK* genes, certain subfamily genes showed substantial structural exon–intron divergences and gene length differences. In genes with fewer introns, increased expression in plants has been previously reported^30,31^. A compact gene structure with few introns has enabled rapid activation and responsiveness to different environmental conditions^31^. However, when the transcriptome data used in this study was combined, we found no evidence that *TaSnRK* gene with fewer introns had shown higher expression levels than the other TaSnRKs genes.

According to accumulating data, gene activity was often linked to discrepancies in the promoter region^32^. In gene promoter regions, *cis*-elements played a major role in controlling gene expression during development and environmental changes^33,34^. TaSnRKs had several *cis*-elements, including growth hormones, MyB, ABRE, and LTR according to promoter analysis in this investigation. Most gene promoters contained at least one of these components, demonstrating that many TaSnRKs were capable of responding to a variety of abiotic stresses while also promoting growth. When gene expression profiles from TaSnRKs with MyB and ABRE *cis*-elements were merged under drought stress, TaSnRKs with MyB and ABRE *cis*-elements increased by an average of 6.3-fold, but TaSnRKs without *cis*-elements only increased by 3.5-fold. As a result, *cis*-elements analysis can help with gene function studies, particularly gene expression patterns under different stress conditions.

*TaSnRK* gene expression levels were analysed using transcriptome data in different tissues and organs of *T. aestivum*^26^. The research results showed that expression patterns of these genes are divided into three categories (Fig. 7). In this study, it we found that subgroup-2 TaSnRKs contain fewer *cis*-elements than TaSnRKs in subgroups-1 and -3 in their promoters. Every gene in subgroup-1 has an average of 5.08 Auxin, 13.55 MeJA, and 8.47 ABRE, 1.69 MYB, 3.38 LTR compared with each gene in subgroup3 and averages of 2.05 in Auxin, 11.08 of MeJA, 1.43 of Gibberellins and 6.67 ABRE, 1.43 MYB, 0.61 LTR, each gene in subgroup 2 with a total of 0.35 Auxin, 9.12 MeJA, 4.56 Gibberellin, 3.50 ABRE, 1.40 MYB, 0.35 LTRE. These data showed that *TaSnRK* activity is linked to promoter region differences.

The roles and functions of several TaSnRKs in response to different abiotic stresses were also determined. Drought stress findings showed that ABA production and *A. thaliana* signals in response to drought were orthologous to *AtSnRK2.3*^39^ and imply that *TaSnRK2.11* and *AtSnRK2.3* play the same role in response to drought stress. The extreme expression changes in *T. aestivum* against effects of drought, salt, thermal stress and ABA induction were reported in *TaSnRK2.8, TaSnRK2.12, TaSnRK3.11, TaSnRK2.16, TaSnRK3.23* and *TaSnRK3.83* whereas its orthologs, *AtSnRK2.2*, could also respond to osmotic stress and ABA induction in *A. thaliana*. This shows that under different conditions the *TaSnRK2.11* gene may be activated substantially.

Previous research has shown that ABA-independent regulation of SnRKs occurs in terrestrial plants, such as Arabidopsis *SnRK2.1, SnRK 2.4, SnRK 2.5, SnRK 2.9*, and *SnRK 2.10*, which are activated by osmotic stress following an ABA-independent pathway^7^. Arabidopsis ABA-independent SnRKs control stress related gene/transcripts under hyperosmotic conditions, complementing the action of ABA-dependent SnRK2s function^35^. As reported in other plants, ABA-independent SnRK2s in wheat also showed sensitive reaction to osmotic stress. Specific responses to low nitrogen or sulfur deprivation^36^, however, appear to be initiated in an ABA-independent manner, as in other plants. These findings support the hypothesis that the plant-specific SnRK2 subfamily is important in stress response signalling both in Arabidopsis and wheat. These pathways are not solely responsible for energy-saving decisions, but they do resulting complex remodelling of cell metabolism, as evidenced by the interactions with DNA repair and maintenance pathways and TOR in Arabidopsis^37^ network as indicated by STRING studies. The fact that a single stress induces the expression of a large number of SnRK2 genes implies that there is a significant compensating impact or pleiotropy within this family in wheat. It is well known that most stresses result in oxidative damage^38,39^. The third SnRK subfamily, the other hand, played an important role in response to osmotic, salt, and heat stresses, because it consists of proteins kinases interacting with calcineurin B-like calcium binding domains^7^ which are mostly involved in drought and salt resistance, being the SOS (salt overly sensitive) mechanism being the best-known^40^.

The findings of this study provide a thorough description of the *SnRK* gene family in *T. aestivum*. It helps us to better understand the biological role of specific *TaSnRK* genes in *T. aestivum*. The study presented just a fundamental characterisation of the *TaSnRK* genes and a comprehensive functional validation would be required to hold the importance of the SnRK family.

## Conclusions

*SnRK* genes are involved in a variety of signalling pathways, including responses to biotic and abiotic stresses. In this study, *SnRK* gene family has been intensively investigated in wheat. A total of 174 *TaSnRK* genes were discovered and categorized into three subgroups based on motif composition and gene structural similarity within each subfamily. Phylogenetic analyses of *SnRK* genes in *A. thaliana* and *O. sativa* can also be used to derive the evolutionary characteristics of the *TaSnRK *genes. Furthermore, the TaSnRK family's microRNA targeting, *cis*-acting elements, and gene expressions were investigated in order to better understanding the biological role of *TaSnRK* genes in *T. aestivum*.

## Materials and methods

### Identification and characterization of SnRKs

Protein sequences of SnRKs identified in related plant species were obtained from the Phytozome database (http://www.phytozome.net/) and the Rice Annotation Project (RAP) (https://rapdb.dna.affrc.go.jp/). BLASTP searches were performed against the bread wheat protein sequences (ftp:/ftp.ensemblgenomes.org/pub/plants/release-51/fasta/triticum aestivum/pep/) using an e-value cut-off of 0.0001 and bit-score > 100. Potential SnRK candidates were discovered using the methods described above. Following the removal of duplicate results, the final sequences were checked for the existence of SnRK related domains using HMMscan (https://www.ebi.ac.uk/Tools/hmmer/search/hmmscan), the SMART database (http://smart.embl-heidelberg.de/)^41^ PFAM^42^ NCBI CDD (http://www.ncbi.nlm.nih.gov/Structure/cdd/wrpsb.cgi)^43^. Additionally, tools from ExPASy (http://www.expasy.ch/tools/pitool.html) were used to compute the number of amino acids, molecular weights (MW), and isoelectric point (pI) of each SnRK protein.

### Phylogenetic analysis of SnRK genes family

A multiple sequence alignment was performed with ClustalW with default settings^44,45^ of 174 non-redundant TaSnRK amino acid. Using MEGA X and the Neighbor-Joining (NJ) technique^45^, a phylogenetic tree was created utilizing the poisson model, pairwise deletion, and 1000 replications of bootstrap. MEGA X has been used to create an unrooted NJ tree that includes all *A. thaliana* and *O. sativa* SnRKs protein sequences.

### Motif composition and gene structural analysis of the SnRK gene family in *T. aestivum*

The Multiple Expectation Maximization for Motif Elicitation (MEME) online tool^46^ (http://meme.sdsc.edu/meme/itro.html) was used to find conserved motifs in TaSnRK proteins with the following parameters: The number of repetitions is unlimited, the maximum number of motifs is ten, and the ideal motif length is six to one hundred residues. The exon–intron structures of TaSnRK family genes were analysed using the Gene Structure Display Server online application (GSDS v.2.0: http://gsds.cbi.pku.edu.ch) based on the gff3 data file^47^.

### Analysis of chromosomal location and orthologous genes in *T. aestivum*

Using MapChart version 3.0^48^, the chromosomal coordinates of all *TaSnRK* genes were mapped to 21 chromosomes of the wheat genome based on physical location from the Plant ensemble database. All *T. aestivum* gene sequences were aligned using BLASTP with an e value of 1e^−10^ to find gene duplication. The pattern of duplicated SnRK were classified as segmental, tandem duplications using MCScanX with default parameters^49^. A tandem duplication is defined as a chromosomal area of less than 200 kb that contains two or more genes^50^. TBtools were used to exhibit synteny relationships of the orthologous *SnRK* genes between *T. aestivum* and *A. thaliana*, the syntenic analysis maps and synonymous (Ks) and non-synonymous (Ka) substitution of each duplicated *TaSnRK* gene^51^.

### *cis*-Elements in promoter regions of TaSnRKs

The wheat genome database was used to extract upstream sequences (1500 bp) from the start codon of each *TaSnRK* gene, which were subsequently utilised for *cis*-element distributions in promoter regions using PlantCARE software (http://bioinformatics.psb.ugent.be/webtools/plantcare/html/)^52^.

### Prediction of MIR genes targeting TaSnRK

The *TaSnRK* gene transcript sequences were obtained from the wheat genome database. The psRNATarget service was used to examine the matured miRNA sequences^53,54^ and the TaSnRK transcript sequences with default settings^55,56^.

### Analysis of protein–protein interactions

The STRING v1054 databases were used to identify protein–protein functional interactions.

SnRK protein sequences were uploaded to the STRING^57^ application, and the database was searched with *A. thaliana* as the reference organism. All identified interaction partners were gathered and searched against the *A. thaliana* genome using blast software at e-value 1−e10.

Using Cytoscape^58^, the one best-hit for each gene was selected for the creation of a PPI network. Finally, the top hub gene from the interaction network was predicted using Cytoscape's cytoHubba plugin (Cytoscape Consortium 2016).

### RNA-seq derived gene expression profiling

The value of TPM (transcripts per million) for each TaSnRK was obtained from the expVIP database (http://www.wheat-expression.com/). Clustvis (https://biit.cs.ut.ee/clustvis/) was used to create heatmaps^59^.

### Plant material and growth conditions

In this experiment, seeds of bread wheat genotypes (C-306, WL-711, RAJ3765, HS240, Kharchia 65, and HD2687) were procured from Germplasm Unit, ICAR-Indian Institute of Wheat and Barley Research, Karnal, India. Seeds were sterilised with 1% sodium hypochloride for 10 min and then rinsed with distilled water three times and germinated in petri plates at 22 °C under controlled conditions. Seedlings were moved to a culture bottle filled with full-strength Hoagland's solution after 5 days of germination and allowed to grow for seven days. Each genotype was seeded in two sets of three biological replications. For drought stress, two contrasting wheat genotypes, C306-drought tolerant and WL711, drought susceptible were used. After 14 days of growth in Hoagland's solution, one set of seedlings was given osmotic shock using 25% (v/v) polyethylene glycol (PEG) 6000 for 24 h, while untreated used as control^60^. Leaf samples from control and stressed seedlings were harvested at above mentioned time intervals for expression analysis. For heat stress, two contrasting wheat genotypes were chosen: RAJ3765-heat resistant and HS240-heat sensitive. These plants were kept at 42 °C for 3 h (at Basal), followed by at 37 °C for 3 h (at Normal), and finally at 42 °C for 3 h (at Acquired). At the time interval stated above, leaf samples from the basal and acquired stress levels were collected. Kharchia 65-salt resistant and HD2687-salt sensitive wheat genotypes were utilised to study salt stress. Two week old seedlings of both genotypes were subjected to 150 mM NaCl treatment. The leaf samples were collected 12 h and 24 h after the treatment. All obtained samples were wrapped in foil and immediately frozen in liquid nitrogen at − 80 °C for total RNA isolation.

### RNA isolation and real time PCR analysis

RNA was extracted using TRIzol Reagent according to the manufacturer's instructions. To get DNA-free RNA, the extracted RNA was treated with DNase I (NEB, USA). The first strand cDNA synthesized from 1 g of total RNA by using Superscript-III reverse transcriptase (Invitrogen, USA). The cDNA was diluted to 1:2 for real-time qRT-PCR analysis, and 1.5 µL of the diluted cDNA was utilised as a template in a 20 µL reaction volume according to the manufacturer's instructions. Real time quantitative RT-PCR analysis was carried out by using the BIO-RAD CFX96 using SYBR Green (Bio-Rad). The endogenous control β-actin was used to standardise the expression data^61^. Using the 2^−ΔΔCt^ method, the expression was measured as a relative fold change^62^. The error bars show the standard deviation of the three biological replicates' expression.

### Research statement

Experimental research and field studies on plants complies with relevant institutional, national, and international guidelines and legislation.

## Supplementary Information

Supplementary Table S1.

Supplementary Table S2.

Supplementary Table S3.

Supplementary Table S4.

Supplementary Table S5.

Supplementary Figures.

Supplementary Legends.
