# Supplementary material for: Genome-wide identification and expression analysis of sucrose nonfermenting-1-related protein kinase (SnRK) genes in Triticum aestivum in response to abiotic stress
Source: Sci Rep. 2021 Nov 18;11:22477. doi: 10.1038/s41598-021-99639-5 (PMC8602265; doi:10.1038/s41598-021-99639-5)
Supplement: Supplementary file 6 — Supplementary Figures. [file 41598_2021_99639_MOESM6_ESM.pptx]

## Slide 1
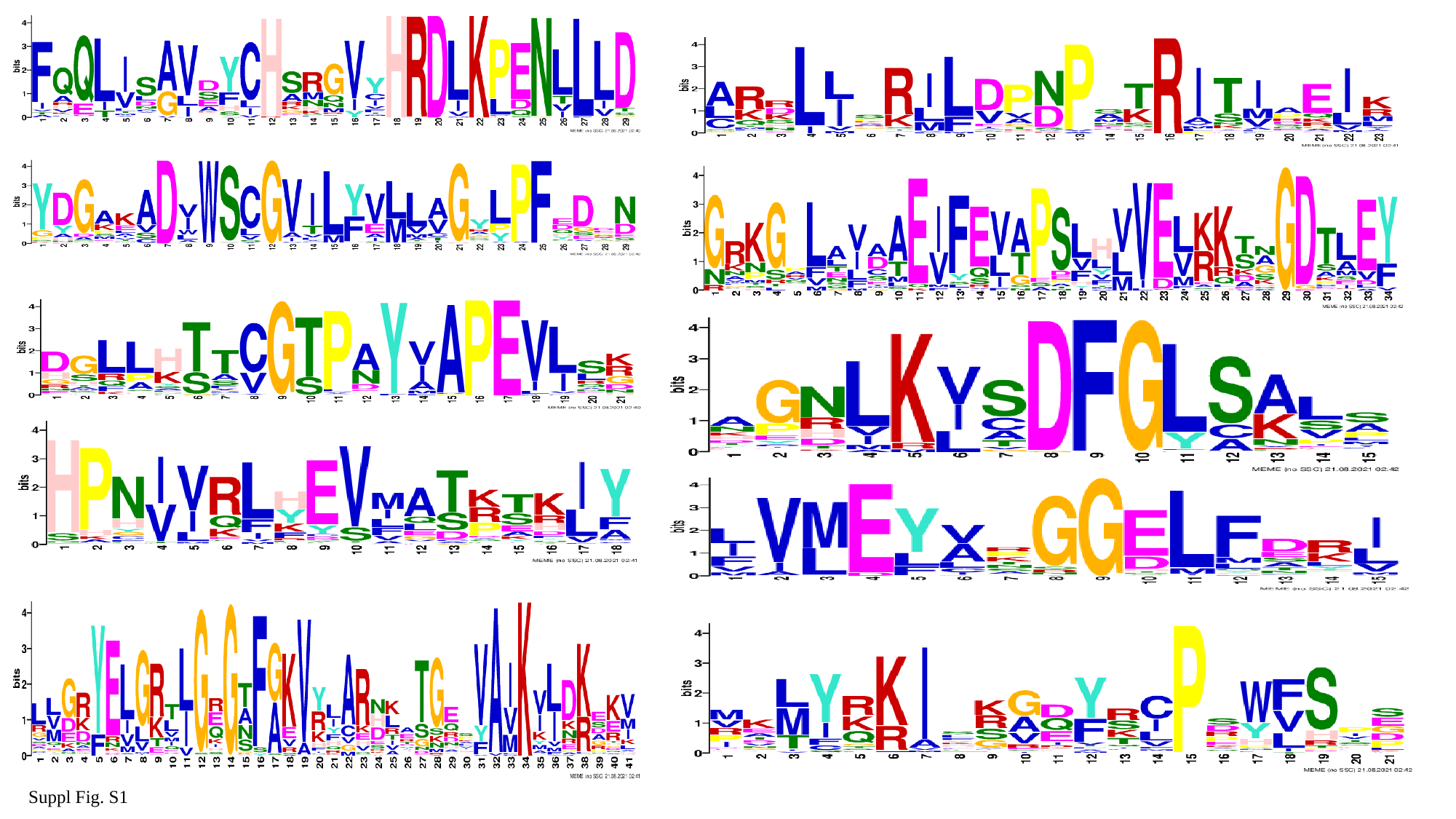

Suppl Fig. S1

## Slide 2
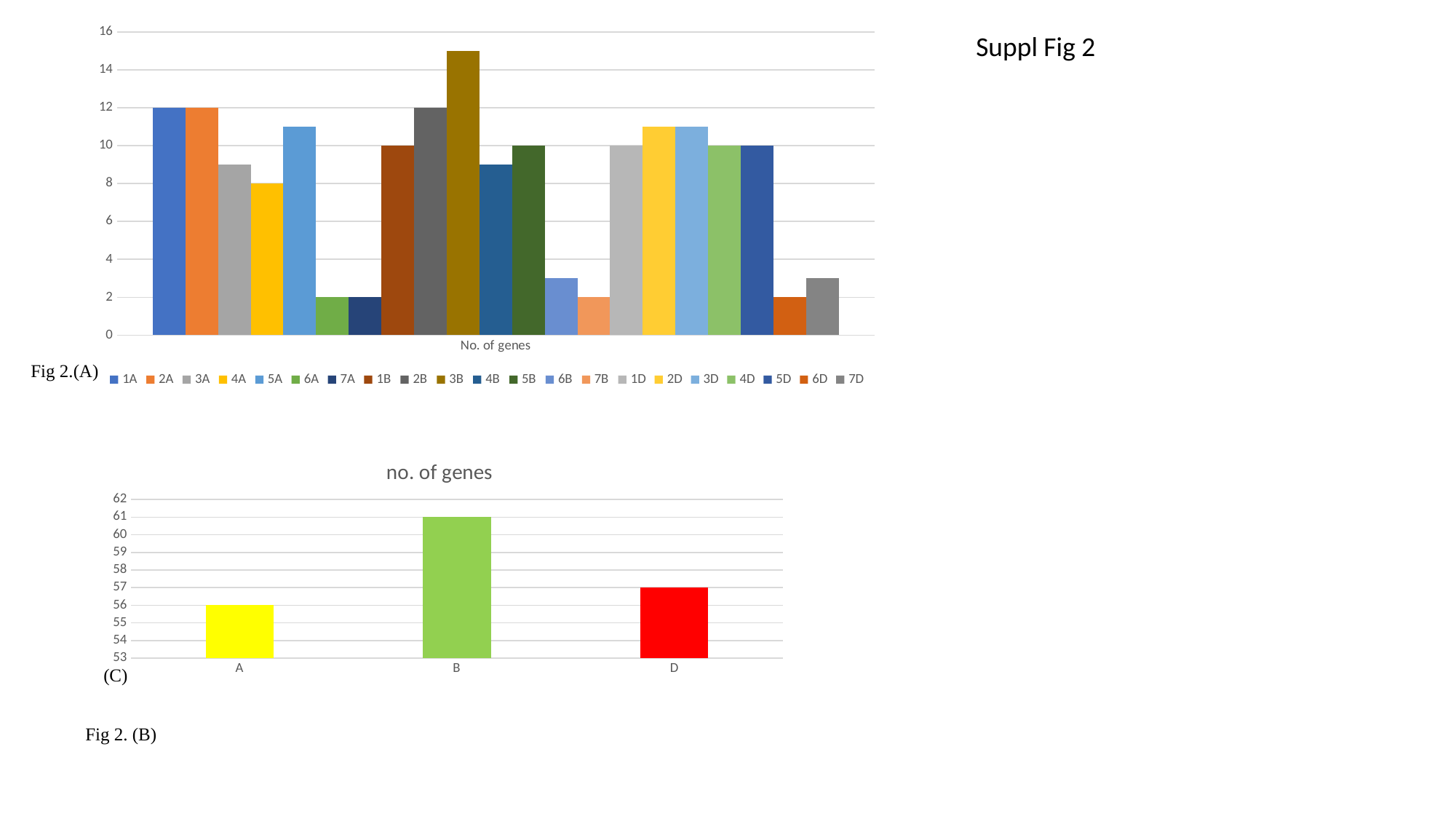

### Chart
| Category | 1A | 2A | 3A | 4A | 5A | 6A | 7A | 1B | 2B | 3B | 4B | 5B | 6B | 7B | 1D | 2D | 3D | 4D | 5D | 6D | 7D |
|---|---|---|---|---|---|---|---|---|---|---|---|---|---|---|---|---|---|---|---|---|---|
| No. of genes | 12.0 | 12.0 | 9.0 | 8.0 | 11.0 | 2.0 | 2.0 | 10.0 | 12.0 | 15.0 | 9.0 | 10.0 | 3.0 | 2.0 | 10.0 | 11.0 | 11.0 | 10.0 | 10.0 | 2.0 | 3.0 |Suppl Fig 2
Fig 2.(A)
### Chart:
| Category | no. of genes |
|---|---|
| A | 56.0 |
| B | 61.0 |
| D | 57.0 |(C)
Fig 2. (B)

## Slide 3
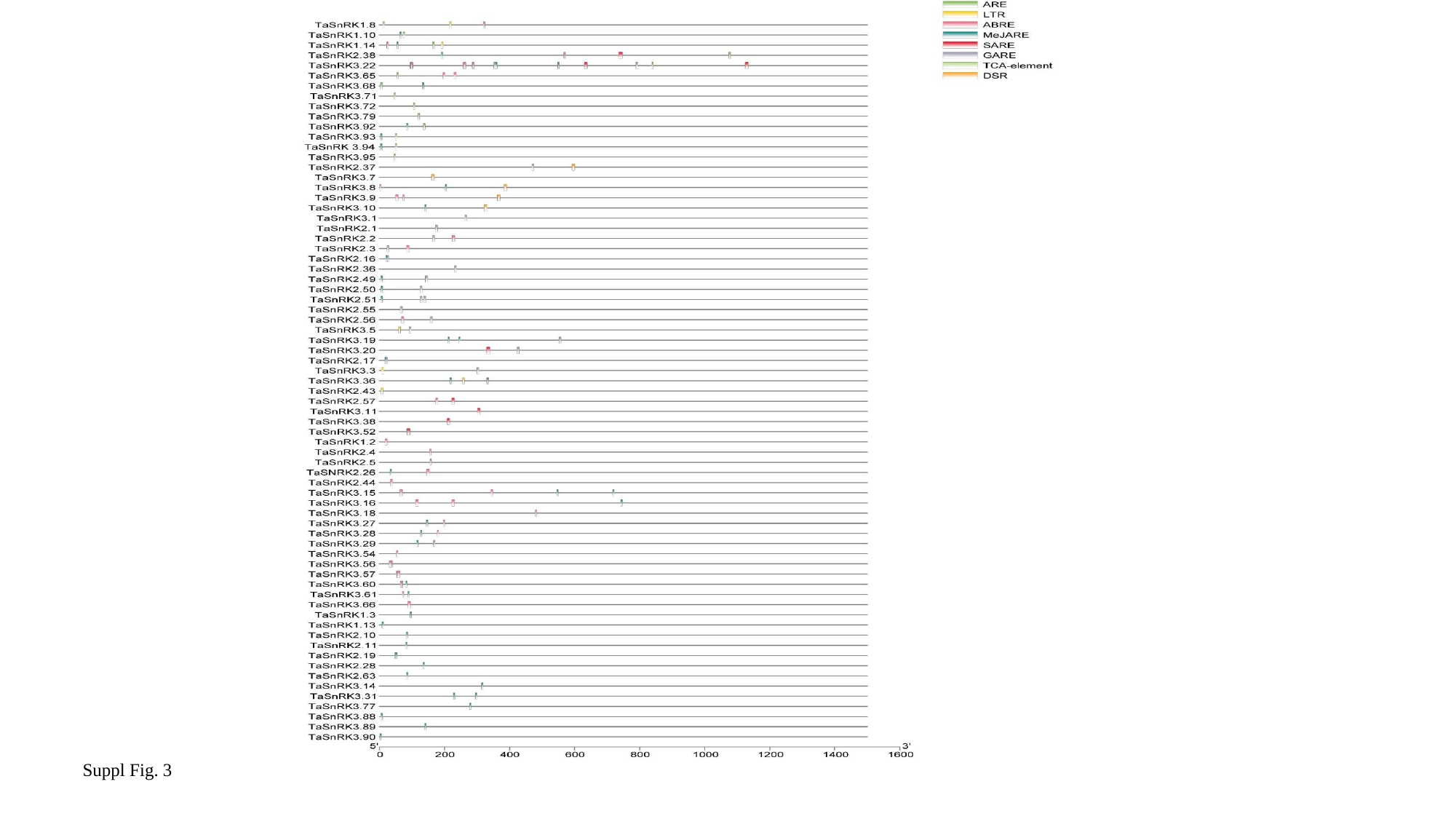

Suppl Fig. 3
