# Supplementary material for: Genome-wide identification and expression analysis of sucrose nonfermenting-1-related protein kinase (SnRK) genes in Triticum aestivum in response to abiotic stress
Source: Sci Rep. 2021 Nov 18;11:22477. doi: 10.1038/s41598-021-99639-5 (PMC8602265; doi:10.1038/s41598-021-99639-5)
Supplement: Supplementary file 7 — Supplementary Legends. [file 41598_2021_99639_MOESM7_ESM.docx]

**Legends of Supplementary figure**

**Fig. 1** Logos showing the conserved residues.

**Fig. 2** (A) Distribution of SnRK genes across 21 chromosomes. (B) Distribution of SnRK genes in the three sub-genomes.

**Fig. 3** *cis*-regulatory elements identified in *TaSnRK* genes. The promoter region (–1500 bp upstream) of *TaSnRK gene* s were scanned for the presence of conserved *cis*-regulatory elements using PlantCare database.

**Supplementary table:**

**Table S1**. Gene ID’s of SnRK genes of Wheat, Rice and Arabidopsis and chromosomal location of genes in wheat genome.

**Table S2**. Detailed information for the 10 motifs in the SnRK proteins of *T. aestivum*.

**Table S3**. Orthologous and paralogous genes of wheat with inter and intra-species.

**Table S4**. Ka/Ks ratio of paralogous genes

**Table S5**. Description of cis-regulatory elements on the basis of upstream region of the genes.
